# Effects of gellan gum and inulin on mixed‐gel properties and molecular structure of gelatin

**DOI:** 10.1002/fsn3.2077

**Published:** 2021-01-24

**Authors:** Juanjuan Wang, Xue Zhao, Changyu Zhou, Chong Wang, Yanyan Zheng, Keping Ye, Chunbao Li, Guanghong Zhou

**Affiliations:** ^1^ Key Laboratory of Meat Processing and Quality Control MOE Key Laboratory of Meat Processing MOA Jiangsu Synergetic Innovation Center of Meat Processing and Quality Control Nanjing Agricultural University Nanjing P.R. China

**Keywords:** gel properties, gelatin, gellan gum, inulin, mixed‐gel

## Abstract

Gellan gum (GG) is often added to gelatin (GL) to improve the gel property. GG‐based or inulin (IL)‐based hydrogels were developed. Rigid and brittle gels or smooth and delicate gels were prepared with GG and IL, respectively. This study aimed to explore the properties and interaction mechanisms of the mixed‐gel system containing GL, GG, and IL, in which different ratios of GG‐IL (0.4%) (10:0, 8:2, 6:4, 5:5, 4:6, 2:8, and 0:10) were added to GL (6%). Texture profiles, rheological properties, water mobility, intermolecular forces, circular dichroism (CD) spectra, and microstructures were analyzed. The results showed that addition of GG‐IL could improve the hardness, chewiness, and cohesiveness of mixed‐gel, besides maintaining appropriate springiness. Water mobility of the mixed‐gel decreased while viscoelasticity increased upon the addition GG. At GG:IL = 2:8, the melting temperature of mixed‐gel was far higher than that of GL gel itself. The GL‐GG‐IL gel showed decrease in nonspecific bonding and increase in hydrogen bonding compared with the GL gel. CD spectra indicated the promotion of GL unfolding by GG, hence suggesting the binding of GG to GL; binding ability was better at GG:IL >5:5. Cryo‐*SEM* provided evidence for the formation of cross‐linked network within GL‐GG‐IL. Overall, we concluded that addition of GG‐IL to GL system would be most suitable for improving the properties of mixed‐gel. This finding may be potentially applicable in the further development of gel food products, such as meat jellies and gummy jellies.

## Graphical abstract

This study aimed to explore the properties and underlying interactions of a mixed‐gel system containing gelatin (GL), gellan gum (GG), and inulin (IL), with different ratios of GG‐IL (10:0, 8:2, 6:4, 5:5, 4:6, 2:8, and 0:10) added to GL. we concluded that addition of GG‐IL into GL system is the most feasible combination for improving the properties of mixed‐gel.

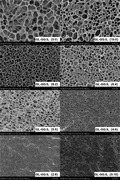

## 1 INTRODUCTION

Proteins and polysaccharides are the most abundant natural biological macromolecules. Most of them have good gel properties, owing to which, they are commonly used as raw materials in food processing. In presence of both, proteins interact with polysaccharides to obtain better properties and improve the texture and stability of food, as is done for enhancing the mouthfeel of meat products (Gibis et al., 2015), jelly products (Witczak et al., 2020), and dairy products (Hussein et al., 2011).

Gelatin (GL) is a polypeptide obtained by hydrolyzing collagen—skin, meat, and bones of animals are rich in collagen (Karim & Bhat, 2008). Owing to its good water‐holding, film‐forming, and biocompatible properties, GL has been widely used in food products. Particularly, GL is the main ingredient of gummy jellies (Jiamjariyatam, 2018), meat jellies (Kim et al., 2020), and edible coatings (Huang et al., 2020). However, GL is a cold‐induced gel. The peptide chains are linked by hydrogen bonds, which are easily broken at a relatively high temperature (Liu et al., 2015). When the temperature reaches only 35–40℃, hydrogen bonds are broken and GL starts to melt (Bello et al., 1956; Djabourov & Papon, 1983; Gilsenan et al., 2003). Therefore, its temperature‐dependent nature limits its applications. For example, the sensory quality of meat jellies is poor due to its easy melting at room temperature (Feiner, 2006), and the applicability of GL edible films is limited due to low thermal stability (Canpean et al., 2013). Considering this shortcoming of GL, many polysaccharides have been added to the GL system till date to improve its stability and processing properties. Benichou et al. (2002) reported that adding polysaccharides produces highly thermal stable protein gels, which can be greater resistant to external handling (high pressure) during food processing. Li et al. (2020) showed that adding polysaccharides improves the properties of "Yu Dong" (a novel protein‐based gel).

Gellan gum (GG) is an anionic polysaccharide produced by *Sphingomonas elodea*, with excellent gel ability and high tolerance to heat during processing (Zia et al., 2018). GG molecules are disorderly curled in aqueous solution during heating and may change to double‐helix structure, induced by intermolecular hydrogen bonding, during cooling. With further lowering of temperature, the double‐helix structures might interact with each other, forming a three‐dimensional network structure, eventually leading to a hard but brittle gel (Morris et al., 2012). Addition of ions can promote GG gelling, since the ions can link GG molecules together by bridging, thereby increasing the gel‐melting temperature and making the system more stable (Osmalek et al., 2018). GG is a commercially available gelling agent. Mixing of GG and GL can increase their application in food industry, as a combination of gelling agents, providing desirable texture, and performing protein–polysaccharide interactions (Banerjee & Bhattacharya, 2011). At present, GG is often added to GL to improve the gel property, which has been studied in the form of gel ( Lau et al., 2000, 2001; Lee et al., 2003).

Inulin (IL) is a natural fructan (polymer) (Meyer et al., 2011); it is a new type of natural soluble dietary fiber that can not only enhance satiety, but also control blood lipid and blood sugar levels, resist tumor formation, improve the intestinal environment, and promote vitamin synthesis and mineral absorption (Shoaib et al., 2016). A certain amount of IL dissolved in water can form a smooth, delicate, and cream‐like gel (Chiavaro et al., 2007) that has the potential to combine with any other hard gel to generate a new gel with improved sensory qualities. Recently, GG, when added to GL, has been shown to improve its gel properties (Kia et al., 2020; Sow et al., 2017). Moreover, IL added to GL, instead of starch, can form slightly softer, springier, and stickier jellies (Delgado & Banon, 2018). However, the characteristics of GG‐IL‐GL mixed‐gel have not yet been reported.

In food production, GL products (such as meat jelly) are often flavored with salt, and GG is often used together with CaCl_2_, to improve the gel properties. In this study, different ratios of GG‐IL (0.4%) (10:0, 8:2, 6:4, 5:5, 4:6, 2:8, and 0:10) were added to GL (6%) containing NaCl (0.6%) and CaCl_2_ (0.2%). Their gel properties were determined by analyzing the texture profiles (TPA), rheological properties, water mobility, intermolecular forces, circular dichroism (CD) spectra, and microstructures. Understanding the protein–polysaccharide (GG‐IL‐GL) interaction can provide a basis for furthering studies toward the development of novel and innovative gel food.

## 2 MATERIALS AND METHODS

### 2.1 Materials

GG (type B) was purchased from Aladdin Biology Co., Ltd. (G108396; Shanghai, China); GL powder was purchased from Phytotech Co., Ltd. (71010‐52‐1); and IL, extracted from chicory with a purity of 90%, was purchased from Sangon Biotech Co., Ltd. (A602227‐0100). All other reagents and chemicals were of analytical grade.

### 2.2 Preparation of sample

Three grams of GL was fully dispersed in 30 g of ultrapure water (UP), and then dissolved well in a water bath with magnetic stirrer (FJS‐2, Fuwei Experimental Instrument Factory) at 75°C for 5 min. GG and IL were added to 17 g and 1 g of UP, respectively, and dissolved similarly using the water bath with magnetic stirrer, at 75°C for 5 min. Next, 0.3 g of NaCl and 0.1 g of CaCl_2_ were dissolved in 1 g of UP. The dissolved NaCl and CaCl_2_ were first added to GL solution and mixed well, after which GG and IL solutions were quickly added. Finally, different groups (GL, 6%, w/w; GG and/or IL, 0.4%, w/w; NaCl, 0.6%, w/w; and CaCl_2_, 0.2%, w/w) were mixed evenly at 75°C. The specific treatment is shown in the Table 1 below.

**TABLE 1:** Specific treatment of samples with the addition of different ratios of GG and IL to GL

Group	GL‐GG:IL (0:0)	GL‐GG:IL (10:0)	GL‐GG:IL (8:2)	GL‐GG:IL (6:4)	GL‐GG:IL (5:5)	GL‐GG:IL (4:6)	GL‐GG:IL (2:8)	GL‐GG:IL (0:10)
GL^a^ + UP^b^/g	3 + 30	3 + 30	3 + 30	3 + 30	3 + 30	3 + 30	3 + 30	3 + 30
NaCl + UP/g	0.3 + 1	0.3 + 1	0.3 + 1	0.3 + 1	0.3 + 1	0.3 + 1	0.3 + 1	0.3 + 1
CaCl_2_ + UP/g	0.1 + 1	0.1 + 1	0.1 + 1	0.1 + 1	0.1 + 1	0.1 + 1	0.1 + 1	0.1 + 1
GG^c^ + UP/g	0 + 17	0.20 + 17	0.16 + 17	0.12 + 17	0.10 + 17	0.08 + 17	0.04 + 17	0 + 17
IL^d^ + UP/g	0 + 1	0 + 1	0.04 + 1	0.08 + 1	0.10 + 1	0.12 + 1	0.16 + 1	0.20 + 1
Total UP/g	50	50	50	50	50	50	50	50

^b^ UP—ultrapure water.

^c^ GG—gellan gum.

^d^ IL—inulin.

^a^ GL—gelatin.

### 2.3 Texture profile analysis (TPA)

TPA was performed according to the method of Huang et al. (2007), with some modifications. The prepared samples were packed into a model cylinder with a diameter of 1.8 cm and height of 2.0 cm, placed at 4°C for 24 hr, and then studied using a texture analyzer (TA.XT Plus; Stable Micro Systems) at room temperature (25°C). The parameters were set as follows: measure type, TPA; probe type, P/50; compression strain, 30%; pretest speed, 2.0 mm/s; test speed, 1.0 mm/s; posttest speed, 1.0 mm/s; and trigger type, auto‐5 g. Based on the characteristics of the samples, data on hardness (g), springiness, chewiness (g), cohesiveness (N), and resilience were recorded and used as the analysis index.

### 2.4 Rheological measurements

#### 2.4.1 Steady shear measurements

Steady shear was measured according to the method of Zhu et al. (2017), with some modifications. The samples were immediately subjected to measurements by a rheometer (MCR 301, Anton Paar), equipped with a steel plate (diameter, 50 mm; gap, 0.5 mm), at 75°C. The steady shear from 0.1 to 1,000 1/s and apparent viscosity were recorded.

#### 2.4.2 Dynamic rheological measurements

Dynamic rheology was studied according to the method of Montero et al. (2002), with some modifications. Pretreatment was the same as in steady shear; temperature sweeps were conducted at a heating rate of 5°C/min at 1 Hz and 5% strain (within the linear viscoelastic range), starting at 75°C for 2 min, then reduced from 75 to 15°C, and finally increased from 15 to 75°C. The values of storage modulus (G′) and loss modulus (G″) were recorded.

### 2.5 Low field nuclear magnetic resonance (LF‐NMR) measurements

LF‐NMR was determined according to the method of Li et al. (2019), with some modifications. Approximately 2 g of gel samples (after being refrigerated at 4°C for 24 hr) in 15‐mm‐diameter glass bottles were used for measuring water mobility and distribution by the Pulsed NMR analyzer (MesoMR23‐060V‐1; Niumag Electric Corporation). The Carr‐Purcell‐Meiboom‐Gill (CPMG) pulse sequence was used to obtain the proton spin‐spin *T*_2_ values at 32°C. The number of echoes was 18,000, number of scans was 4, duration between successive scans (*T*_W_) was 5,000 ms, and time of echoes was 0.4 ms. The exponential decay graph was obtained by inversion. And the water mobility and distribution were analyzed by the relaxation time value and the peak areas percentage.

### 2.6 Intermolecular force measurements

Gels (after being refrigerated at 4°C for 24 hr) were solubilized in the following chemicals, selected for their capacity to cleave specific bonds: 0.05 M NaCl (SA), 0.6 M NaCl (SB), 0.6 M NaCl + 1.5 M urea (SC), and 0.6 M NaCl + 8 M urea (*SD*) (Liu et al., 2011). Two grams of chopped gel were homogenized in 10 ml of the above solutions at 5,000 rpm for 2 min (PD 5000‐TP; PRIMA). The resulting homogenates were stirred at 4°C for 1 hr, and then centrifuged at 20,000 *g* for 15 min (Avanti J‐E; Beckman Coulter). Protein concentration in supernatants was determined by BCA protein assay kit (PC0020; Solarbio Science & Technology Co., Ltd).

Proteins were partially solubilized in the above solutions to measure the amount of nonspecific bonds (SA), ionic bonds (difference between SB and SA), hydrogen bonds (difference between SC and SB), and hydrophobic interactions (difference between *SD* and SC). Results are expressed as "mg soluble protein/mL of homogenate."

### 2.7 CD spectral analysis

CD spectroscopy was performed according to the method of Qi et al. (2018). The diluted samples (0.3 mg/ml) were loaded in a 1‐mm‐pathlength quartz cell and CD spectrum recorded in a Chirascan spectrometer (J‐1500; JASCO Corporation). The parameters were set as follows: wavelength range, 185–260 nm; scan rate, 100 nm/min; path length, 0.1 cm. Ultrapure water was used as blank to obtain the mean residue ellipticity (θ).

### 2.8 Cryogenic scanning electron microscopy (Cryo‐*SEM*)

The prepared samples were refrigerated at 4°C for 24 hr before the experiment. The microstructure of gels was analyzed using a cryogenic scanning electron microscope (SU8010, Hitachi, Japan) equipped with PP3010T Cryo‐*SEM* preparation system. Approximately 1–3‐mm^3^ gel was placed into a sample retainer and rapidly frozen in liquid nitrogen (−210°C). The frozen gel samples were transferred to a refrigerated transport unit (−150°C) (PP3010T; Quorum), fractured with a razor blade, and sublimed at − 70°C for 10 min to remove ice from the surface of the gels. Finally, samples were gold coated by sputtering with ultrapure argon at the accelerating voltage of 15 kV, and experiments performed at −150°C. The magnification of microstructural images was 10,000×.

### 2.9 Statistical analysis

All experiments were conducted with at least three replicates. The data were analyzed by one‐way analysis of variance, using SAS 8.1 (SAS Institute Inc.). Duncan's multiple‐range test (*p* < .05) was used to compare the significant differences between the results. The data are expressed as mean ± standard deviation and processed by Origin Pro 8 (OriginLab Inc.).

## 3 RESULTS

### 3.1 TPA results

According to TPA (Table 2), both hardness and springiness of the mixed‐gel with only GG added were significantly affected compared with that in the control group (0:0), (*p* < .05). Hardness of the mixed‐gel (10:0) reached a maximum value of 1,250.73 g, which was 7.48 times higher than that of the control group, whereas springiness significantly decreased. Hardness of the mixed‐gel with only IL added (0:10) decreased (*p* < .05), along with its springiness, which was, however, higher than that of the 10:0 group (*p* < .05). This suggested GG as the determinant of mixed‐gel hardness, as is consistent with previous reports (Lau et al., 2000; Sow et al., 2017), possibly owing to the formation of new "heterolytic junction zones" by GG and GL (Pranoto et al., 2007). GG is often added to gummy candy products to strengthen their gel structure; however, the addition of GG alone would make it too hard, thereby affecting the mouthfeel (Kia et al., 2020; Moritaka et al., 1999). Moreover, adding GG to GL could significantly increase the chewiness of mixed‐gel (*p* < .05). With the addition of IL, springiness of the mixed‐gel was maintained at 0.94, its cohesiveness increased, and chewiness decreased (*p* < .05). Previous results from our laboratory had shown the addition of IL to improve mouthfeel and sensory score of pig skin jelly. Delgado and Banon (2018) had also reported IL (90 g/kg raw mass) addition into GL to generate slightly softer, springier, and stickier jellies than starch. These results together suggested the addition of GG‐IL into GL to improve the sensory quality of related products to some extent, which could further be related to the increased hardness and chewiness caused by the addition of GG, and increased cohesiveness caused by the addition of IL. Therefore, addition of GG or IL alone to GL could not achieve the desired mouthfeel, whereas addition of GG‐IL could enhance the texture and sensory qualities of the mixed gel. Furthermore, IL could also enrich the functionalities of gel products.

**TABLE 2:** TPA of gels with different ratios of GG‐IL added to GL

Group	Hardness (g)	Springiness	Chewiness (g)	Cohesiveness (*N*)	Resilience
GL^a^‐GG^b^:IL^c^ (0:0)	167.10 ± 12.56^g^	0.97 ± 0.02^a^	146.89 ± 11.47^g^	0.91 ± 0.01^b^	0.85 ± 0.01^a^
GL‐GG:IL (10:0)	1,250.73 ± 74.30^a^	0.93 ± 0.02^c^	720.70 ± 35.62^a^	0.61 ± 0.03^g^	0.36 ± 0.02^g^
GL‐GG:IL (8:2)	891.08 ± 33.54^b^	0.94 ± 0.01^b^	573.60 ± 11.46^b^	0.68 ± 0.02^f^	0.42 ± 0.02^f^
GL‐GG:IL (6:4)	633.79 ± 22.31^c^	0.94 ± 0.02^c^	442.84 ± 16.39^c^	0.75 ± 0.01^e^	0.50 ± 0.01^e^
GL‐GG:IL (5:5)	485.50 ± 18.03^d^	0.94 ± 0.03^c^	368.18 ± 18.75^d^	0.81 ± 0.01^d^	0.59 ± 0.02^d^
GL‐GG:IL (4:6)	365.80 ± 32.21^e^	0.94 ± 0.03^c^	284.74 ± 22.64^e^	0.83 ± 0.03^c^	0.61 ± 0.03^c^
GL‐GG:IL (2:8)	213.88 ± 10.02^f^	0.96 ± 0.02^c^	184.37 ± 11.84^f^	0.91 ± 0.00^b^	0.77 ± 0.01^b^
GL‐GG:IL (0:10)	130.66 ± 13.33^h^	0.95 ± 0.03^b^	114.86 ± 10.83^h^	0.94 ± 0.00^a^	0.86 ± 0.02^a^

Note

Means in the same column with different superscript letters indicate significant differences (*p* < .05).

^a^ GL—gelatin.

^c^ IL—inulin.

^b^ GG—gellan gum.

### 3.2 Rheological analyses

#### 3.2.1 Steady shear analyses

**FIGURE 1:**
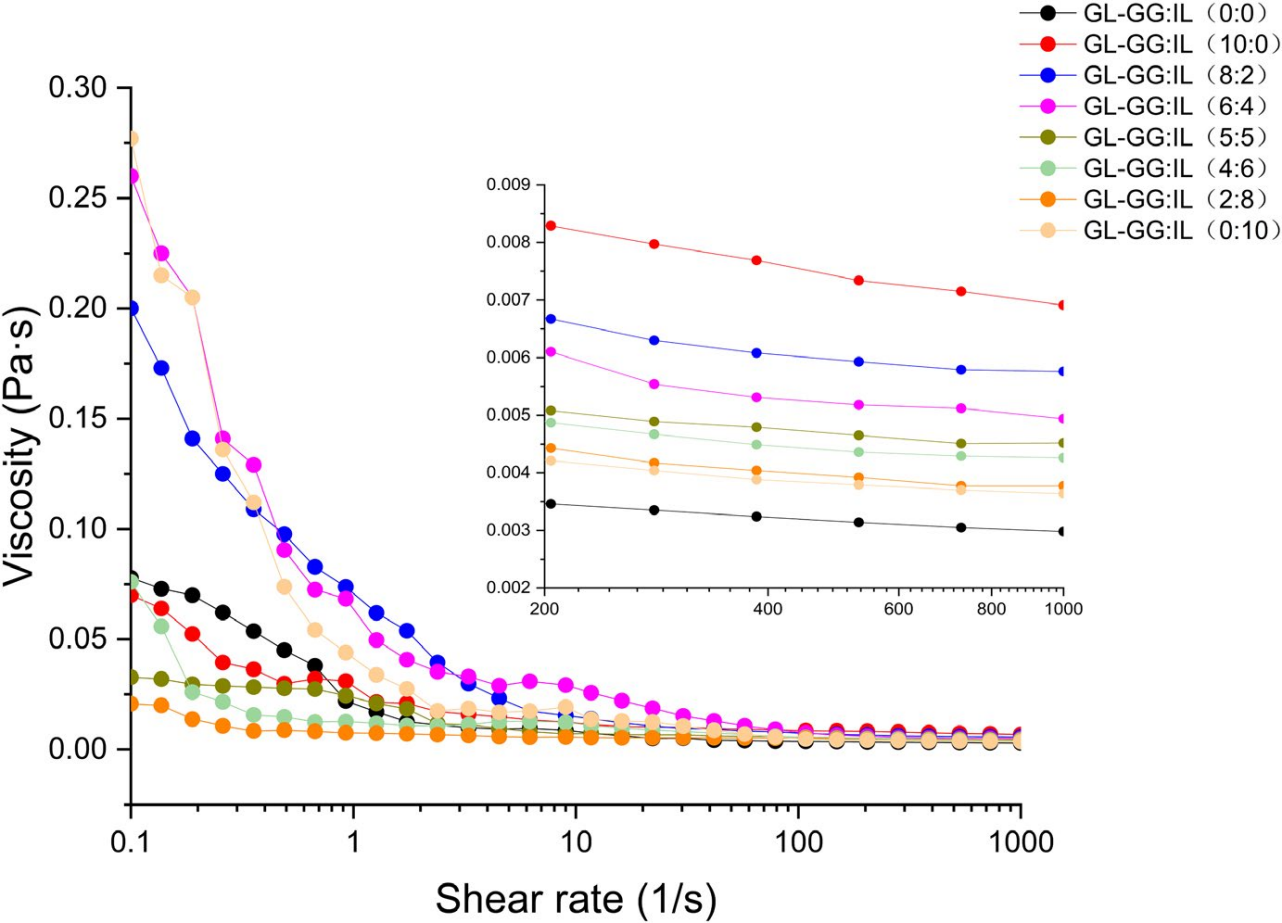
Changes in viscosity of gels with different ratios of GG‐IL added to GL

Viscosity is a macroscopic indicator that reflects the microstructure; the shear thinning behavior is mainly due to the destruction of intramolecular or intermolecular interactions under shear action (Grisel & Muller, 1998). Figure 1 shows the viscosity changes in different groups. The viscosity of all samples decreased with increase of shear rate, indicating shear thinning behavior in the control and treatment groups; all samples were typical nonNewtonian fluids. The partially enlarged view in Figure 1 shows the control group (0:0) to have the lowest viscosity. When different ratios of GG‐IL were added to GL, viscosity of the mixed‐gels increased; the greater the ratio of GG, the greater was the viscosity of mixed‐gel. Zhuang et al. (2019) had observed that adding insoluble dietary fiber (polysaccharide) to myofibrillar protein emulsion gels could increase the viscosity of gel system, implying that dietary fiber absorbed water from the mixing system, increased the relative concentration of myofibrillar protein, and enhanced molecular cross‐linking between protein molecules. This observation suggested that addition of GG‐IL could enhance the interactions in mixed‐gel system and promote the formation of gel network.

#### 3.2.2 Dynamic rheological analyses

**FIGURE 2:**
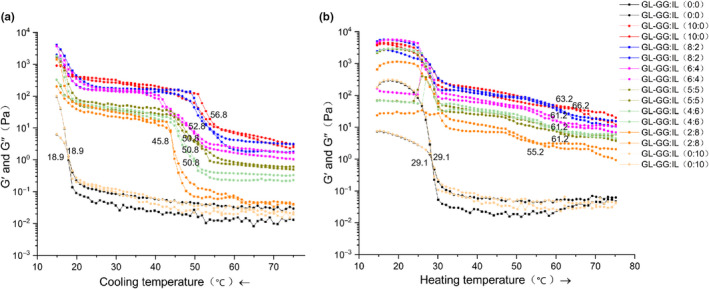
Changes in dynamic viscoelastic behavior of samples due to the addition of different ratios of GG‐IL to GL; (a) G' and G" during cooling, (b) G' and G" during heating

Dynamic rheological properties of the gel, during cooling, are displayed in Figure 2a, plotted in terms of elastic modulus (G') and viscous modulus (G"). The G' and G" of all samples increased with decrease in temperature, hence indicating the interaction between macromolecules in the mixed‐gel system (such as hydrogen bonds) to change during cooling (Nieto‐Nieto et al., 2015), gradually leading to the increase of viscoelasticity and formation of a three‐dimensional network structure of the gel. Figure 2b shows G' and G" during heating. When the gels were heated, G' and G" of all samples decreased, indicating that high temperature could transform the gel structure from an ordered three‐dimensional network structure to a disordered state. Moreover, irrespective of cooling or heating, the viscoelasticity of treatment groups was higher than that of the control group; higher the ratio of GG, greater was the viscoelasticity, which was consistent with the steady rheological results.

In dynamic viscoelasticity measurement, the temperature at which G' begins to exceed G" during cooling is generally defined as the gelling temperature, and the temperature at which G" begins to exceed G' during heating is defined as the melting temperature (Dai et al., 2020). The gelling temperature, when IL alone was added (0:10), was around 18.9°C, and the melting temperature was 29.1°C, both of which being consistent with the control group (0:0) (*p* > .05), thereby indicating that IL could not change the gelling and melting temperatures of GL. However, the addition of GG significantly improved the gelling and melting temperatures of the mixed‐gel; for example, the gelling temperature after adding GG alone (10:0) was 56.8°C while the melting temperature was 66.2°C, which may be related to the structural characteristics of the two polysaccharides. In the presence of Ca^2+^, the electrostatic repulsion between carboxyl side chains of GG molecules was shielded, which formed bridges between pairs of carboxyl groups on neighboring helices and showed a more stable three‐dimensional network structure. Thus, the gelling and melting temperatures were increased (Huang et al., 2004). For the treatment group of GG:IL = 2:8, both gelling and melting temperatures decreased evidently, although they were still higher than those of the control group (0:0), with the gelling temperature approximately 45.8°C and melting temperature approximately 55.2°C.

### 3.3 Water mobility

**FIGURE 3:**
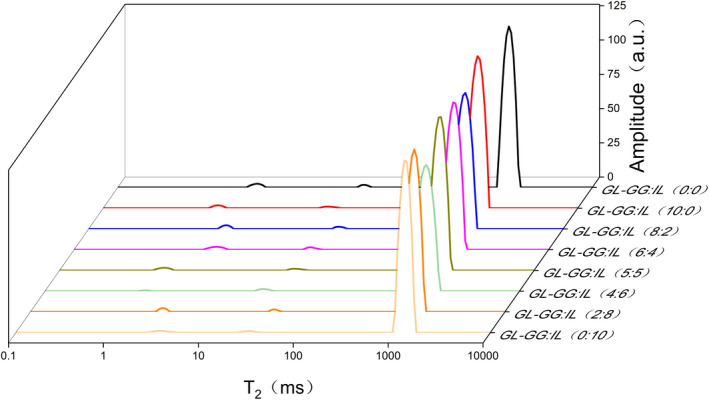
CPMG relaxation time of gels in the range of 0.1–10000 ms after adding different ratios of GG‐IL to GL

Change in spin‐spin relaxation time (*T*_2_) can characterize the mobility and distribution of water in gels; shorter the initial *T*_2_, stronger would be the bond between water molecules and the substrates (Shaarani et al., 2006). As shown in Figure 3, *T*_2_ peaks correspond to three states of water, namely, the worst mobile water (*T*_2b_), weakly mobile water (*T*_21_), and free water (*T*_22_) (Liu, Cai, et al., 2019). The range of *T*_2b_, *T*_21_, and *T*_22_ are 1–10, 10–100, and 100–10000 ms, respectively. The signal intensity of *T*_22_ was far greater than that of *T*_2b_ and *T*_21_, which clearly suggested free water to be an important component of the mixed‐gel system. Similar water mobility and distribution have previously been observed in starch gel (Chen et al., 2017).

The relaxation time (*T*_22_) and peak areas percentage (*P*_22_) of free water are listed in Table 3. In the control group (0:0), *T*_22_ was 907.53 ms; when only GG (10:0) or IL (0:10) was added, *T*_22_ was 678.95 ms and 906.71 ms, respectively, indicating that GG caused shortening of *T*_22_, while IL did not. Therefore, with increasing ratio of IL, *T*_22_ value increased, though lower than that in the control group. This phenomenon may be because GL and GG can interact to form more hydrogen bonds, thus, promoting the formation of gel network structure and restricting the mobility of free water. In general, water content is proportional to the peak area of relaxation curve (Tang et al., 2000). However, in this study, *P*_22_ showed no significant difference between the groups (*p* > .05), suggesting that no water exchange occurred. Similar result was observed in κ‐carrageenan and ε‐polylysine mixed‐gels (Li et al., 2019). Therefore, the addition of GG‐IL in different ratios can limit the mobility of water, promote combination between the protein–polysaccharide and water, and make the gel structure more stable. Formation mechanism of the GL‐GG‐IL mixed‐gel was further analyzed by exploring the intermolecular forces, CD spectra, and microstructure.

**TABLE 3:** Relaxation time (*T*_22_) and peak areas percentage (*P*_22_) of gels with different ratios of GG‐IL added to GL

Group	*T*_22_(ms)	*P*_22_
GL^a^‐GG^b^:IL^c^ (0:0)	907.53 ± 53.98^a^	96.94 ± 1.00^a^
GL‐GG:IL (10:0)	678.95 ± 22.67^d^	97.61 ± 0.92^a^
GL‐GG:IL (8:2)	678.95 ± 22.67^d^	97.02 ± 1.45^a^
GL‐GG:IL (6:4)	719.39 ± 24.02^a^	97.01 ± 1.47^a^
GL‐GG:IL (5:5)	740.20 ± 22.04^c^	96.81 ± 1.51^a^
GL‐GG:IL (4:6)	751.22 ± 22.04^c^	97.52 ± 0.61^a^
GL‐GG:IL (2:8)	819.32 ± 23.36^b^	97.94 ± 0.33^a^
GL‐GG:IL (0:10)	906.71 ± 30.28^a^	96.86 ± 0.86^a^

Note

Means in the same column with different superscript letters indicate significant differences (*p* < .05).

^a^ GL—gelatin.

^b^ GG—gellan gum.

^c^ IL—inulin.

### 3.4 Analyses of intermolecular forces

**FIGURE 4:**
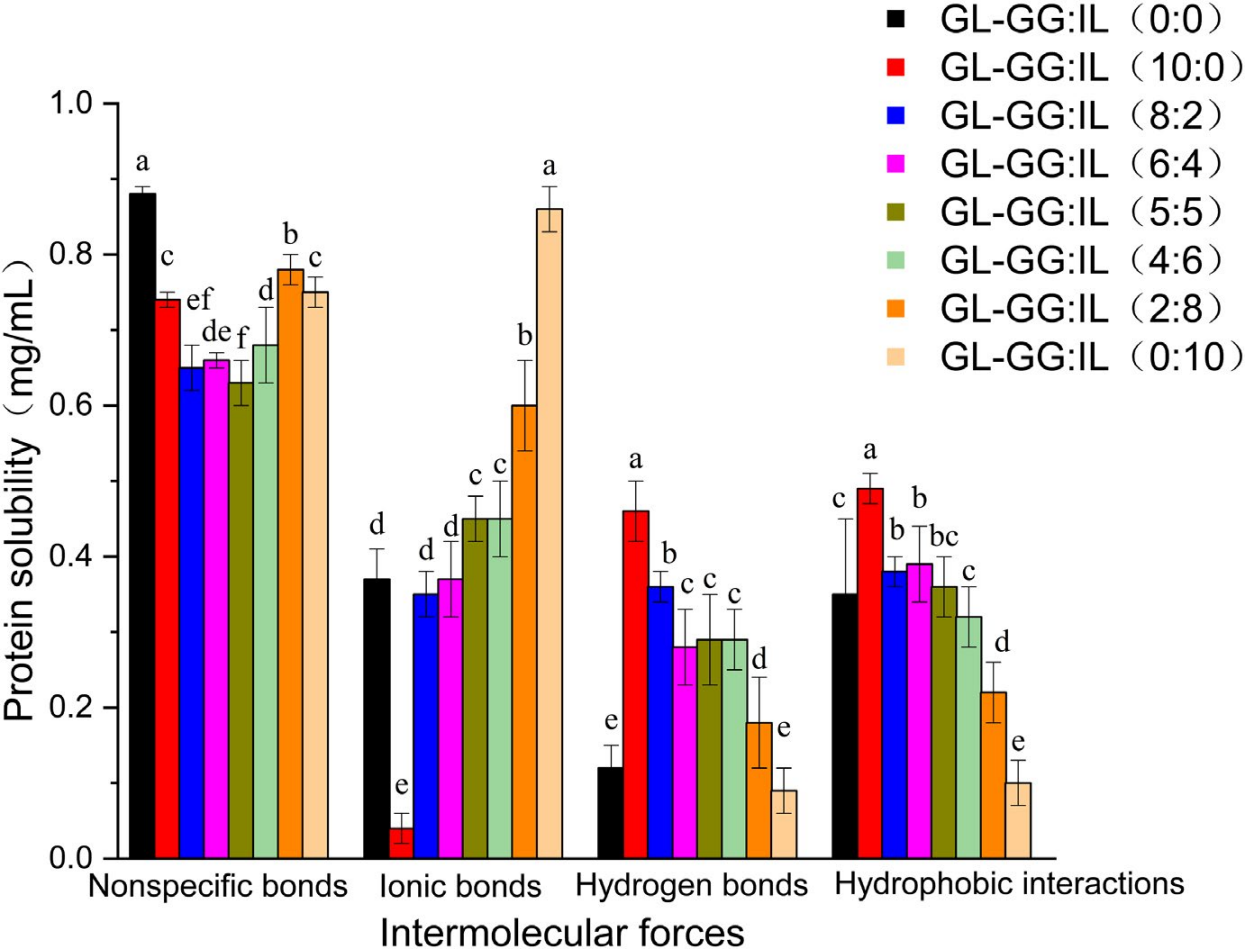
Analyses of the intermolecular forces in gels with different ratios of GG‐IL added to GL. Error bars with different lowercase letters indicate significant differences (*p* < .05)

The protein–polysaccharide mixed‐gel was formed by intermolecular interactions, the strength of which may be characterized by protein solubility; greater the solubility, stronger the intermolecular forces (Tan et al., 2010). Intermolecular forces in the gels were evaluated as shown in Figure 4. Addition of GG and/or IL decreased the nonspecific bonds, indicating both polysaccharides to have specific cross‐linking with proteins (covalent cross‐linking). When GG‐IL was added, the specific cross‐linking was enhanced. Since GG can combine with ions of the gel system to promote gelling, addition of GG alone (10:0) decreased ionic bonds, whereas addition of IL alone (0:10) increased the same. When different ratios of GG‐IL were added to GL, ionic bonds of the GL‐GG‐IL treatment group were similar to those of the control group (0:0), probably due to the offset effect of GG and IL. For hydrogen bonds, adding GG alone (10:0) could increase their number while adding IL alone (0:10) had no significant effect, which was consistent with the result of LF‐NMR. When different ratios of GG‐IL were added to GL, hydrogen bonds were higher than that in the control group (*p* < .05). Previous studies had shown hydrogen bonds and electrostatic interactions (ionic bonds) to be the main chemical forces in the process of protein–polysaccharide gel formation (Imeson et al., 1977; Ustunol et al., 1992). The polysaccharide structure of edible gum contains lots of hydroxyl groups, which can form additional hydrogen bonds with water molecules, and promote the formation of more stable gel network structure (Foegeding & Ramsey, 1987). This suggested GG had an excellent ability to combine with water. The change in hydrophobic interactions after adding GG alone (10:0) was similar to that in hydrogen bonds, and the treatment group (10:0) had a significantly higher hydrophobic effect than the control group (0:0) (*p* < .05). As the ratio of IL increased, hydrophobic interactions of the mixed‐gel decreased, and finally, were lower than that of the control group (0:0) (*p* < .05). These results, regarding hydrophobic interactions, suggested that addition of GG promoted GL unfolding, while addition of IL promoted GL aggregation, which was further confirmed by CD spectra, as discussed in the following section. Overall, GG or IL combined with GL with different intermolecular forces. The structure of a mixed‐gel is usually determined by multiple interactive forces (Asiyanbi et al., 2017; Gilsenan et al., 2003), all of which had influence on the formation of GL‐GG‐IL mixed‐gel. Compared with the control group, the mixed‐gel formed by GL‐GG‐IL mainly showed decrease of nonspecific bonds and increase of hydrogen bonds.

### 3.5 CD spectral analyses

**FIGURE 5:**
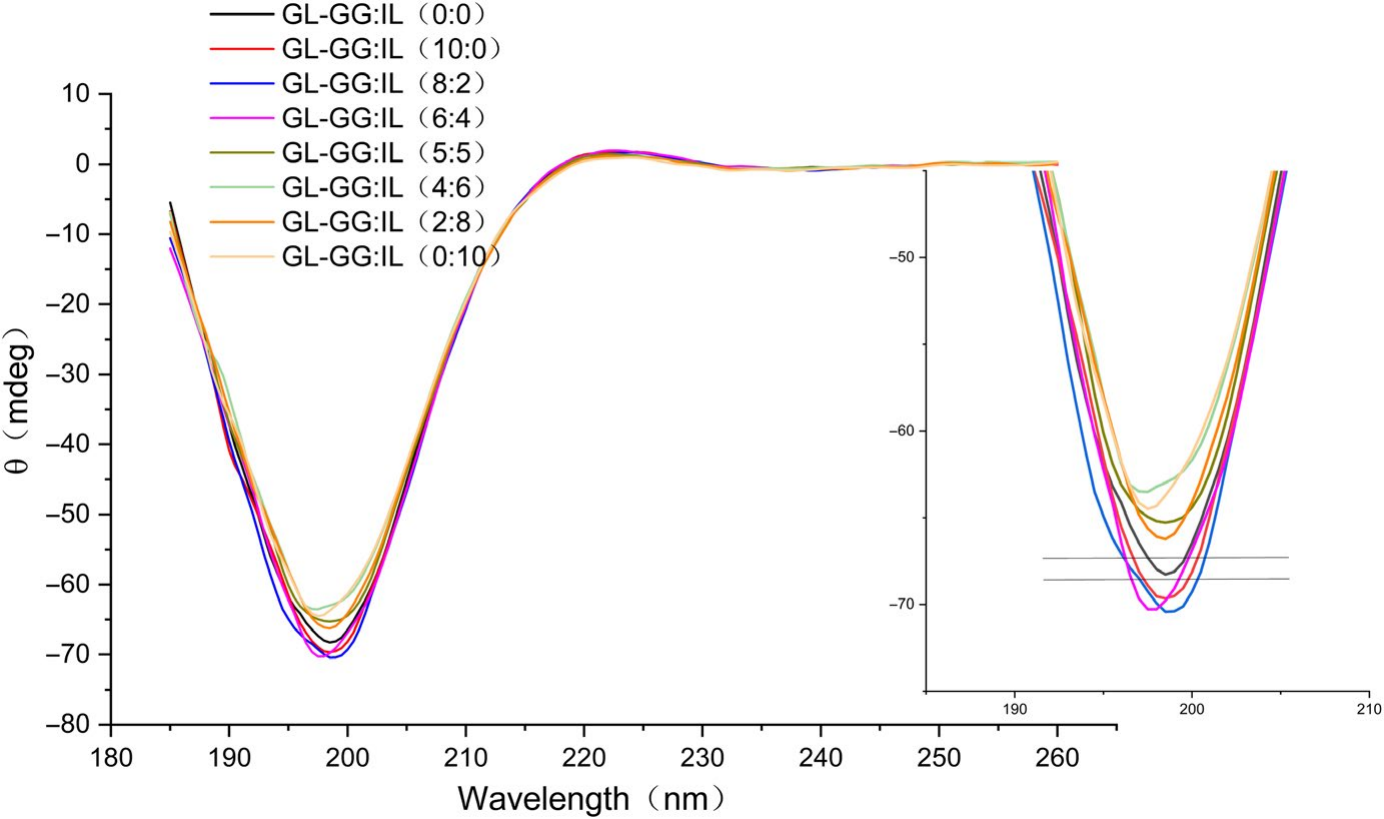
CD spectral analyses of gels with different ratios of GG and IL added to GL

CD spectroscopy is the most rapid and sensitive technique to evaluate the conformational changes of proteins in dilute solution; it can reflect the secondary structure of proteins or polypeptides based on their spectra in the far‐ultraviolet wavelength region (Gopal et al., 2012). From the CD spectrum in Figure 5, two peaks are obvious, namely, the positive peak at 220 nm and the negative peak at 199 nm, similar to that in previous reports, and characteristic of the CD spectra of collagen or gelatin (Li et al., 2009; Losso & Ogawa, 2014); the negative peak at 199 nm represents aggregation of GL molecules (Gómez‐Guillén et al., 2002; Qi et al., 2018). Compared with the control group (located between the two lines), the negative peak value increased when GG alone was added (10:0) and decreased when IL alone was added (0:10), thus, indicating that GG prevented aggregation of GL, and IL promoted the aggregation of GL. Therefore, GG was speculated to reduce the degree of GL aggregation and promote protein unfolding, thereby contributing to the binding of polysaccharide and protein. When the addition of GG was higher than that of IL (GG:IL >5:5), the degree of GL unfolding was higher than that of the control group, that is, the treatment groups with GG:IL >5:5 were more conducive to improving the properties of mixed‐gels.

### 3.6 Microstructure analyses

**FIGURE 6:**
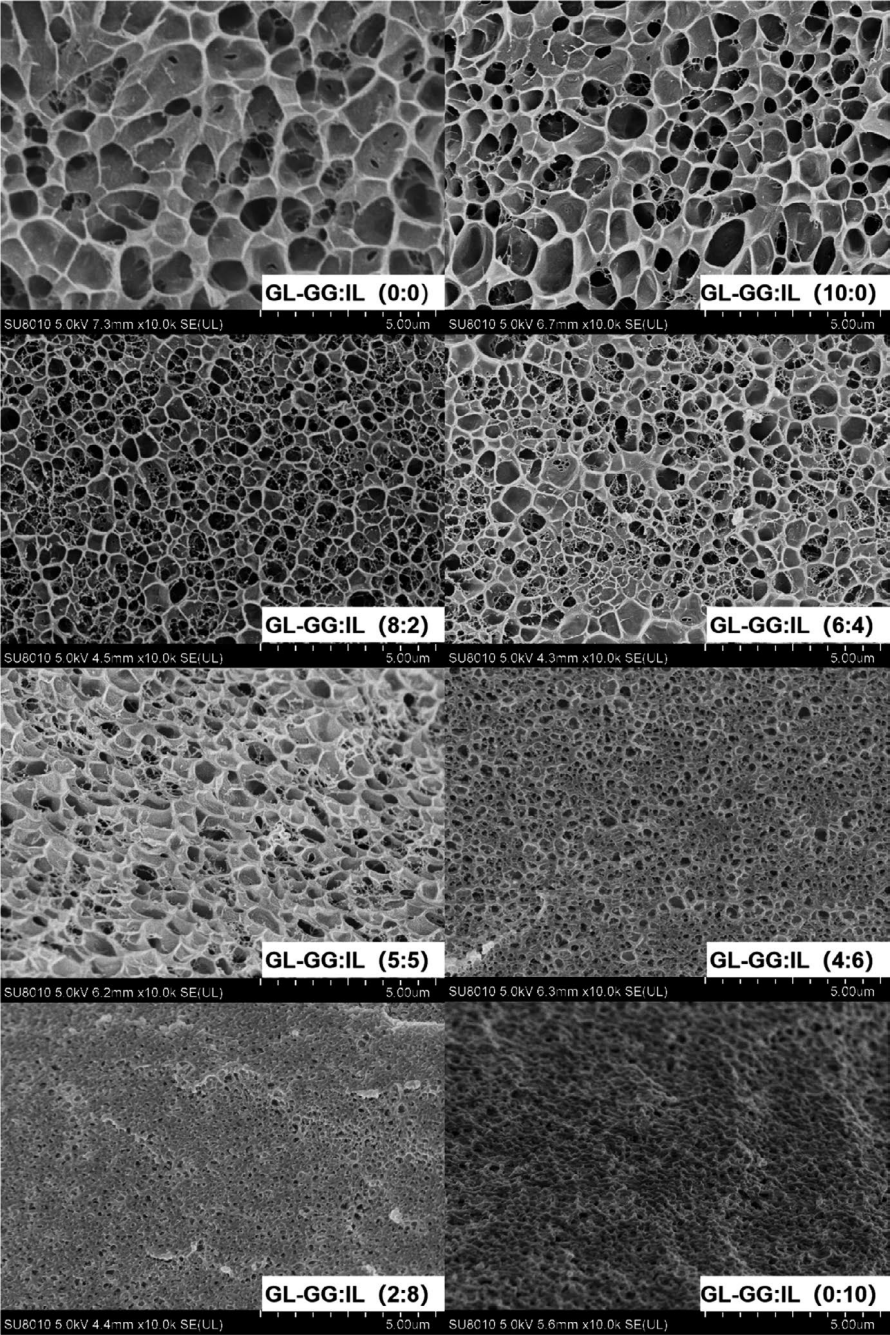
Cryo‐*SEM* micrographs of gels with different ratios of GG‐IL added to GL

Cryo‐*SEM* is an effective technique for characterizing the microstructure of hydrogels; it can reduce the formation of ice crystals in the gel and maintain the original state of the sample maximally, to observe a more accurate spatial structure (Sriamornsak et al., 2008). The microstructural differences across the groups were quite evident (Figure 6). In the control group (0:0), the microstructure was uniform with cavities arranged in order, which mainly resulted from the cross‐linking between GL molecules due to the formation of hydrogen bonds. However, the cavities were larger than in treatment groups, owing to which GL had better springiness and lesser hardness, macroscopically. Addition of GG caused the mixed‐gel cavities to become smaller, indicating that the interaction of GG with GL made the mixed‐gel structure more compact (Petcharat & Benjakul, 2017), which was in good agreement with the results of TPA and rheology. With addition of IL more than GG (GG:IL <5:5), the microstructure of gels was significantly different from that of control group and other treatment groups, showing the network structures formed by GL and/or GG to be coated and filled with IL. For example, when IL was added alone (0:10), the polygonal porous network structure of GL itself was not changed, although it was denser, with IL filling into the cavities. This phenomenon suggested that IL did not establish direct interactions with GL or GG and was primarily only physically embedded in the gelatin matrix, thereby making the rheological and other macroscopic indices similar to those of the control group. The interaction and microstructure of IL and oat protein (Nieto‐Nieto et al., 2015) had been reported previously. Although IL was not incorporated in the oat protein network, it led to denser and more homogeneous protein networks. In this study, the interpretation had been further supported by CD spectroscopy, demonstrating that GG and IL influenced protein unfolding and aggregation, eventually forming different network structures. Several studies have shown that while a small amount of IL can improve the gel properties of porcine myosin or oat protein, a high concentration of IL could have a negative effect (Nieto‐Nieto et al., 2015; Zhang et al., 2020). A similar result was obtained in this study; the addition of IL alone could not promote the formation of a more stable network structure of GL, although the latter was formed when IL was added in combination with GG. Therefore, IL or GG was concluded to cross‐link with GL in distinct ways, and the mixed‐gel system, using the combination of GG‐IL, could form a gel with excellent properties.

## 4 CONCLUSIONS

Two polysaccharides (GG and IL), at different ratios, were dissolved and mixed into GL containing NaCl and CaCl_2_, imparting a significant effect on the properties of the mixed‐gel. GG‐IL, added at different ratios, changed the intermolecular forces of the mixed‐gel system, mainly manifesting as a decrease of nonspecific bonding and increase of hydrogen bonds. Addition of polysaccharides changed the degree of aggregation of GL molecules, such that it was conducive to the combination of polysaccharides and GL at GG:IL >5:5. Changes in the intermolecular forces and molecular structure affected the cross‐link between protein and polysaccharide, ultimately influencing the gel properties, including improved texture, restricted free water mobility, and improved rheological properties, with increased gelling and melting temperatures. Microstructure analysis demonstrated that the GL‐GG‐IL gel could cross‐link effectively with each other. Therefore, compared with adding GG or IL alone, adding different ratios of GG‐IL to GL was shown to effectively improve the gel properties. The mixed use of GL‐GG‐IL should be considered during gel food production; for example, the quality of gummy jellies and meat jellies may be improved by mixing GL‐GG‐IL.

## 5 ETHICAL REVIEW

This study does not involve any human or animal testing.

## CONFLICT OF INTEREST

The authors declare that they do not have any conflict of interest.
